# Tetra­aqua­bis[(1-carboxyl­atomethyl-1,3-benzimidazol-3-ium-3-yl)acetato-κ*O*]palladium(II) dihydrate

**DOI:** 10.1107/S1600536808016097

**Published:** 2008-06-07

**Authors:** Lujiang Hao, Chunhua Mu, Ridong Wang

**Affiliations:** a College of Food and Biological Engineering, Shandong Institute of Light Industry, Jinan 250353, People’s Republic of China; b Maize Research Insitute, Shandong Academy of Agricultural Science, Jinan 250100, People’s Republic of China; c Department of Clinical Medicine, Medical School, Shandong University, Jinan 250012, People’s Republic of China

## Abstract

In the title compound, [Pd(C_11_H_9_N_2_O_4_)_2_(H_2_O)_4_]·2H_2_O, the palladium(II) cation lies on an inversion centre and is hexa­coordinated by two carboxyl­ate O atoms from two (1-carboxyl­atomethyl-1,3-benzimidazol-3-ium-3-yl)acetate ligands and four water mol­ecules, with a slightly distorted octa­hedral geometry. O—H⋯O hydrogen bonds link the mol­ecules together.

## Related literature

For uses of carboxylic acids in materials science, see: Church & Halvorson (1959 ▶). For uses in biological systems, see: Chung *et al.* (1971 ▶); Okabe & Oya (2000 ▶); Serre *et al.* (2005 ▶); Pocker & Fong (1980 ▶); Scapin *et al.* (1997 ▶); Kim *et al.* (2001 ▶).
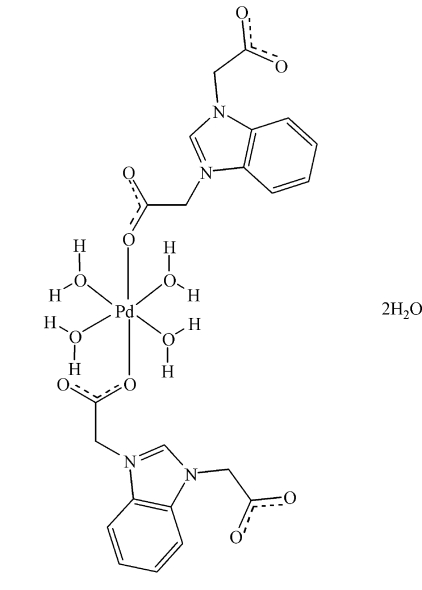


## Experimental

### 

#### Crystal data

[Pd(C_11_H_9_N_2_O_4_)_2_(H_2_O)_4_]·2H_2_O
*M* *_r_* = 680.90
Monoclinic, 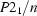

*a* = 5.4702 (10) Å
*b* = 11.794 (2) Å
*c* = 20.886 (3) Å
β = 95.13 (3)°
*V* = 1342.1 (4) Å^3^
*Z* = 2
Mo *K*α radiation
μ = 0.77 mm^−1^
*T* = 293 (2) K
0.43 × 0.28 × 0.22 mm

#### Data collection

Bruker APEXII CCD area-detector diffractometer
Absorption correction: multi-scan (*SADABS*; Bruker, 2004 ▶) *T*_min_ = 0.733, *T*_max_ = 0.849
7075 measured reflections
2425 independent reflections
1958 reflections with *I* > 2σ(*I*)
*R*_int_ = 0.032

#### Refinement

*R*[*F* ^2^ > 2σ(*F* ^2^)] = 0.028
*wR*(*F* ^2^) = 0.072
*S* = 1.00
2425 reflections
205 parameters
9 restraints
H atoms treated by a mixture of independent and constrained refinement
Δρ_max_ = 0.24 e Å^−3^
Δρ_min_ = −0.49 e Å^−3^

### 

Data collection: *APEX2* (Bruker, 2004 ▶); cell refinement: *SAINT-Plus* (Bruker, 2004 ▶); data reduction: *SAINT-Plus*; program(s) used to solve structure: *SHELXS97* (Sheldrick, 2008 ▶); program(s) used to refine structure: *SHELXL97* (Sheldrick, 2008 ▶); molecular graphics: *SHELXTL* (Sheldrick, 2008 ▶); software used to prepare material for publication: *SHELXTL*.

## Supplementary Material

Crystal structure: contains datablocks global, I. DOI: 10.1107/S1600536808016097/cf2196sup1.cif

Structure factors: contains datablocks I. DOI: 10.1107/S1600536808016097/cf2196Isup2.hkl

Additional supplementary materials:  crystallographic information; 3D view; checkCIF report

## Figures and Tables

**Table 1:** Hydrogen-bond geometry (Å, °)

*D*—H⋯*A*	*D*—H	H⋯*A*	*D*⋯*A*	*D*—H⋯*A*
O7—H1*W*⋯O4^i^	0.822 (10)	1.985 (14)	2.756 (3)	156 (3)
O7—H2*W*⋯O5^i^	0.821 (10)	1.937 (10)	2.747 (3)	169 (3)

Symmetry code: (i) 
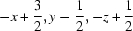
.

**Fig. 1.**
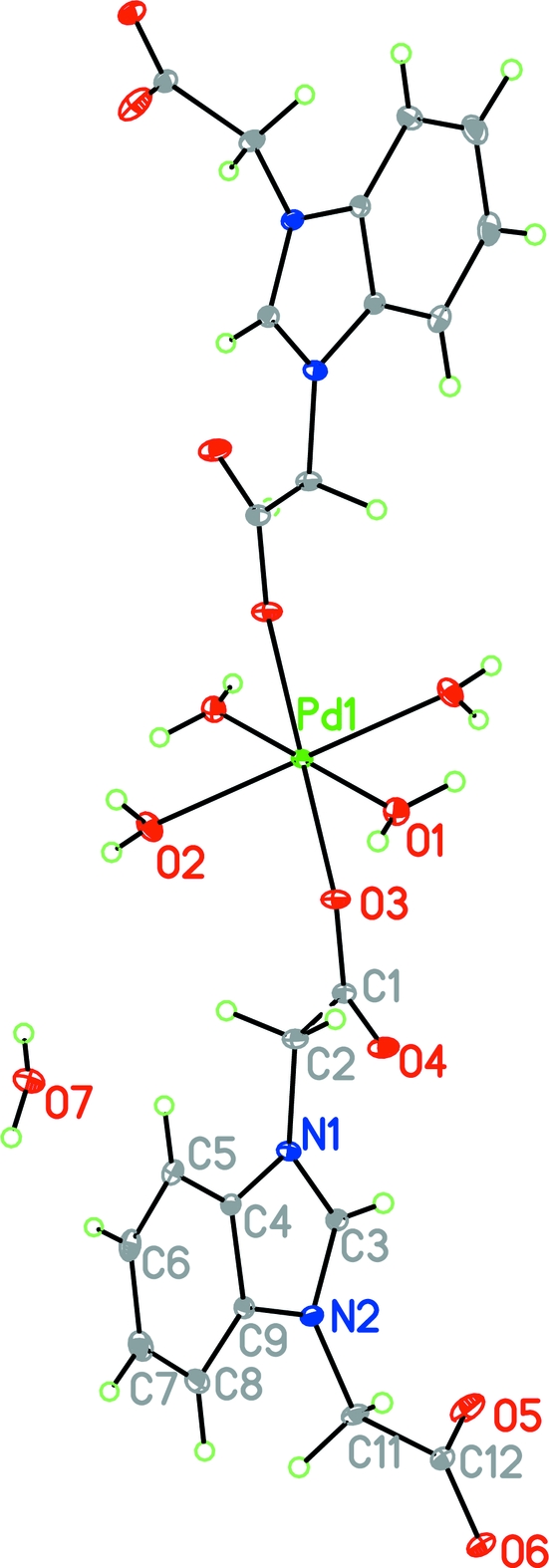
The molecular structure of (I), showing the atomic numbering scheme and 30% probability displacement ellipsoids. [Symmetry code for unlabelled atoms: 1-x, 2-y, -z.]

**Fig. 2.**
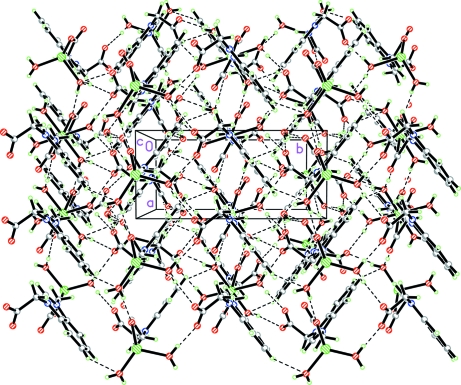
The packing of (I) with hydrogen bonds shown as dashed lines.

## supplementary crystallographic information

### Comment

In recent years, carboxylates have been widely used as polydentate ligands, which can coordinate to transition or rare earth ions yielding complexes with interesting properties that are useful in materials science (Church & Halvorson, 1959; Chung *et al.*, 1971) and in biological systems (Okabe & Oya, 2000; Serre *et al.*, 2005; Pocker & Fong, 1980; Scapin *et al.*, 1997). For example, Kim *et al.* (2001) focused on the syntheses of transition metal complexes containing benzenecarboxylate and rigid aromatic pyridine ligands in order to study their electronic conductivity and magnetic properties. The importance of transition metal dicarboxylate complexes motivated us to pursue synthetic strategies for these compounds, using sodium 1-carboxymethyl-1,3-benzimidazol-3-ium-3-acetate as a polydentate ligand. Here we report the synthesis and X-ray crystal structure analysis of the title compound.

The molecular structure of the title compound is shown in Fig. 1. The palladium(II) cation lies on an inversion center and is hexacoordinated by two carboxylate oxygen atoms from two 1-carboxymethyl-1,3-benzimidazol-3-ium-3-acetato ligands and four water molecules, with a slightly distorted octahedral geometry. The Pd—O bond distances are in the range 2.2608 (19)–2.276 (2) Å. The packing involves hydrogen bonds, shown in Table 1 and Figure 2.

### Experimental

A mixture of palladium dichloride (0.5 mmol), imidazole (1.0 mmol), sodium 1-carboxymethyl-1,3-benzimidazol-3-ium-3-acetate (0.5 mmol), water (8 ml) and ethanol (8 ml) in a 25 ml Teflon-lined stainless steel autoclave was kept at 413 K for three days. Colorless crystals were obtained after cooling to room temperature with a yield of 27%. Anal. Calc. for C_22_H_30_N_4_O_14_Pd: C 38.77, H 4.41, N 8.22%; Found: C 38.68, H 4.37, N 8.14%.

### Refinement

The H atoms of the water molecule were located in a difference density map and were refined with distance restraints H···H = 1.38 (2) Å, O—H = 0.88 (2) Å, and with a fixed *U*_iso_ of 0.80 Å^2^. All other H atoms were placed in calculated positions with a C—H bond distance of 0.93 Å and *U*_iso_(H) = 1.2*U*_eq_(C).

### Crystal data

**Table:**

[Pd(C_11_H_9_N_2_O_4_)_2_(H_2_O)_4_]·2H_2_O	*F*_000_ = 696
*M**_r_* = 680.90	*D*_x_ = 1.685 Mg m^−^^3^
Monoclinic, *P*2_1_/*n*	Mo *K*α radiation λ = 0.71073 Å
Hall symbol: -P 2yn	Cell parameters from 2425 reflections
*a* = 5.4702 (10) Å	θ = 2.0–25.2º
*b* = 11.794 (2) Å	µ = 0.77 mm^−^^1^
*c* = 20.886 (3) Å	*T* = 293 (2) K
β = 95.13 (3)º	Block, colorless
*V* = 1342.1 (4) Å^3^	0.43 × 0.28 × 0.22 mm
*Z* = 2	

### Data collection

**Table:**

Bruker APEXII CCD area-detector diffractometer	2425 independent reflections
Radiation source: fine-focus sealed tube	1958 reflections with *I* > 2σ(*I*)
Monochromator: graphite	*R*_int_ = 0.032
*T* = 293(2) K	θ_max_ = 25.2º
φ and ω scans	θ_min_ = 2.0º
Absorption correction: multi-scan(SADABS; Bruker, 2004)	*h* = −6→6
*T*_min_ = 0.733, *T*_max_ = 0.849	*k* = −13→14
7075 measured reflections	*l* = −24→13

### Refinement

**Table:**

Refinement on *F*^2^	Secondary atom site location: difference Fourier map
Least-squares matrix: full	Hydrogen site location: inferred from neighbouring sites
*R*[*F*^2^ > 2σ(*F*^2^)] = 0.028	H atoms treated by a mixture of independent and constrained refinement
*wR*(*F*^2^) = 0.072	*w* = 1/[σ^2^(*F*_o_^2^) + (0.042*P*)^2^] where *P* = (*F*_o_^2^ + 2*F*_c_^2^)/3
*S* = 1.00	(Δ/σ)_max_ = 0.005
2425 reflections	Δρ_max_ = 0.24 e Å^−^^3^
205 parameters	Δρ_min_ = −0.49 e Å^−^^3^
9 restraints	Extinction correction: none
Primary atom site location: structure-invariant direct methods	

### Special details

**Table:**

Geometry. All e.s.d.'s (except the e.s.d. in the dihedral angle between two l.s. planes) are estimated using the full covariance matrix. The cell e.s.d.'s are taken into account individually in the estimation of e.s.d.'s in distances, angles and torsion angles; correlations between e.s.d.'s in cell parameters are only used when they are defined by crystal symmetry. An approximate (isotropic) treatment of cell e.s.d.'s is used for estimating e.s.d.'s involving l.s. planes.
Refinement. Refinement of *F*^2^ against ALL reflections. The weighted *R*-factor *wR* and goodness of fit *S* are based on *F*^2^, conventional *R*-factors *R* are based on *F*, with *F* set to zero for negative *F*^2^. The threshold expression of *F*^2^ > σ(*F*^2^) is used only for calculating *R*-factors(gt) *etc*. and is not relevant to the choice of reflections for refinement. *R*-factors based on *F*^2^ are statistically about twice as large as those based on *F*, and *R*- factors based on ALL data will be even larger.

### Fractional atomic coordinates and isotropic or equivalent isotropic displacement parameters (Å^2^)

**Table:**

	*x*	*y*	*z*	*U*_iso_*/*U*_eq_	
Pd1	0.5000	1.0000	0.0000	0.02449 (11)	
C1	0.7711 (5)	1.0484 (2)	0.14648 (12)	0.0299 (6)	
C2	0.5544 (5)	0.9924 (2)	0.17543 (12)	0.0326 (6)	
H2A	0.4065	1.0354	0.1631	0.039*	
H2B	0.5316	0.9166	0.1578	0.039*	
C3	0.4539 (5)	1.0371 (2)	0.28620 (12)	0.0294 (6)	
H3	0.3237	1.0858	0.2745	0.035*	
C4	0.7676 (4)	0.9208 (2)	0.28035 (12)	0.0279 (6)	
C5	0.9568 (5)	0.8536 (2)	0.26202 (14)	0.0365 (7)	
H5	0.9834	0.8433	0.2190	0.044*	
C6	1.1029 (6)	0.8030 (3)	0.31070 (17)	0.0480 (8)	
H6	1.2346	0.7586	0.3006	0.058*	
C7	1.0587 (6)	0.8166 (3)	0.37470 (17)	0.0540 (9)	
H7	1.1595	0.7793	0.4062	0.065*	
C8	0.8726 (6)	0.8828 (3)	0.39307 (15)	0.0449 (7)	
H8	0.8444	0.8916	0.4360	0.054*	
C9	0.7282 (5)	0.9361 (2)	0.34427 (12)	0.0299 (6)	
C11	0.4355 (5)	1.0558 (3)	0.40327 (13)	0.0369 (7)	
H11A	0.4276	0.9955	0.4346	0.044*	
H11B	0.2696	1.0831	0.3923	0.044*	
C12	0.5910 (5)	1.1527 (2)	0.43369 (13)	0.0347 (6)	
H1W	0.545 (5)	0.681 (2)	0.2365 (7)	0.042*	
H2W	0.571 (4)	0.693 (2)	0.1719 (9)	0.042*	
H3W	0.160 (5)	1.1619 (14)	0.0196 (13)	0.042*	
H4W	0.074 (4)	1.078 (2)	0.0568 (12)	0.042*	
H5W	0.503 (3)	0.7896 (18)	0.0589 (13)	0.042*	
H6W	0.270 (3)	0.799 (2)	0.0290 (13)	0.042*	
N1	0.5889 (4)	0.98516 (17)	0.24523 (11)	0.0290 (5)	
N2	0.5306 (4)	1.01001 (17)	0.34575 (11)	0.0301 (5)	
O1	0.1897 (3)	1.09974 (17)	0.03739 (10)	0.0394 (5)	
O2	0.3926 (4)	0.83292 (17)	0.04423 (10)	0.0451 (5)	
O3	0.7682 (3)	1.03890 (18)	0.08641 (9)	0.0384 (5)	
O4	0.9339 (4)	1.09448 (18)	0.18150 (9)	0.0460 (5)	
O5	0.7726 (4)	1.1842 (2)	0.40763 (11)	0.0650 (7)	
O6	0.5172 (3)	1.19231 (18)	0.48365 (9)	0.0439 (5)	
O7	0.4821 (4)	0.7010 (2)	0.20114 (10)	0.0485 (5)	

### Atomic displacement parameters (Å^2^)

**Table:**

	*U*^11^	*U*^22^	*U*^33^	*U*^12^	*U*^13^	*U*^23^
Pd1	0.02495 (17)	0.02873 (18)	0.01952 (16)	−0.00361 (11)	0.00047 (11)	0.00121 (11)
C1	0.0280 (14)	0.0368 (15)	0.0252 (15)	0.0003 (12)	0.0035 (11)	0.0001 (12)
C2	0.0323 (15)	0.0429 (17)	0.0225 (14)	−0.0058 (12)	0.0013 (12)	−0.0005 (11)
C3	0.0301 (14)	0.0302 (14)	0.0285 (15)	−0.0017 (11)	0.0057 (12)	−0.0005 (11)
C4	0.0297 (14)	0.0259 (14)	0.0280 (14)	−0.0057 (11)	0.0025 (11)	−0.0017 (11)
C5	0.0348 (15)	0.0316 (16)	0.0433 (18)	−0.0014 (12)	0.0053 (13)	−0.0082 (13)
C6	0.0394 (17)	0.0315 (17)	0.072 (2)	0.0073 (13)	−0.0003 (16)	−0.0058 (15)
C7	0.055 (2)	0.042 (2)	0.061 (2)	0.0064 (16)	−0.0148 (17)	0.0110 (16)
C8	0.0555 (19)	0.0411 (18)	0.0369 (17)	−0.0030 (15)	−0.0020 (14)	0.0067 (13)
C9	0.0336 (15)	0.0261 (15)	0.0295 (15)	−0.0037 (12)	0.0007 (11)	0.0001 (11)
C11	0.0397 (16)	0.0461 (18)	0.0264 (15)	−0.0038 (14)	0.0115 (12)	−0.0088 (13)
C12	0.0360 (15)	0.0362 (16)	0.0322 (16)	0.0023 (12)	0.0049 (12)	−0.0056 (12)
N1	0.0282 (12)	0.0365 (14)	0.0227 (12)	−0.0006 (9)	0.0046 (9)	0.0024 (9)
N2	0.0342 (13)	0.0327 (13)	0.0239 (12)	−0.0027 (9)	0.0058 (10)	−0.0037 (9)
O1	0.0368 (11)	0.0391 (12)	0.0445 (13)	−0.0023 (9)	0.0158 (9)	0.0021 (9)
O2	0.0349 (12)	0.0429 (13)	0.0558 (14)	−0.0055 (9)	−0.0058 (10)	0.0164 (10)
O3	0.0353 (11)	0.0585 (13)	0.0212 (11)	−0.0095 (9)	0.0023 (8)	−0.0004 (9)
O4	0.0425 (12)	0.0675 (15)	0.0279 (11)	−0.0240 (10)	0.0021 (9)	−0.0049 (9)
O5	0.0650 (16)	0.0750 (17)	0.0596 (15)	−0.0360 (13)	0.0314 (13)	−0.0339 (13)
O6	0.0475 (13)	0.0510 (14)	0.0339 (11)	0.0038 (9)	0.0067 (9)	−0.0152 (9)
O7	0.0458 (13)	0.0635 (15)	0.0366 (13)	0.0001 (11)	0.0067 (10)	0.0083 (11)

### Geometric parameters (Å, °)

**Table:**

Pd1—O1	2.2608 (19)	C6—C7	1.389 (4)
Pd1—O1^i^	2.2608 (19)	C6—H6	0.930
Pd1—O3	2.2687 (19)	C7—C8	1.364 (5)
Pd1—O3^i^	2.2687 (19)	C7—H7	0.930
Pd1—O2^i^	2.276 (2)	C8—C9	1.383 (4)
Pd1—O2	2.276 (2)	C8—H8	0.930
C1—O4	1.227 (3)	C9—N2	1.392 (3)
C1—O3	1.258 (3)	C11—N2	1.455 (3)
C1—C2	1.529 (4)	C11—C12	1.528 (4)
C2—N1	1.456 (3)	C11—H11A	0.970
C2—H2A	0.970	C11—H11B	0.970
C2—H2B	0.970	C12—O5	1.232 (3)
C3—N2	1.316 (4)	C12—O6	1.243 (3)
C3—N1	1.329 (3)	O1—H3W	0.832 (10)
C3—H3	0.930	O1—H4W	0.823 (10)
C4—C9	1.383 (3)	O2—H5W	0.828 (10)
C4—C5	1.384 (4)	O2—H6W	0.824 (10)
C4—N1	1.394 (3)	O7—H1W	0.822 (10)
C5—C6	1.373 (4)	O7—H2W	0.821 (10)
C5—H5	0.930		
			
O1—Pd1—O1^i^	180.00 (9)	C5—C6—H6	119.2
O1—Pd1—O3	94.14 (7)	C7—C6—H6	119.2
O1^i^—Pd1—O3	85.86 (8)	C8—C7—C6	122.4 (3)
O1—Pd1—O3^i^	85.86 (8)	C8—C7—H7	118.8
O1^i^—Pd1—O3^i^	94.14 (7)	C6—C7—H7	118.8
O3—Pd1—O3^i^	180.0	C7—C8—C9	116.3 (3)
O1—Pd1—O2^i^	85.34 (7)	C7—C8—H8	121.8
O1^i^—Pd1—O2^i^	94.66 (7)	C9—C8—H8	121.8
O3—Pd1—O2^i^	88.61 (7)	C8—C9—C4	121.6 (3)
O3^i^—Pd1—O2^i^	91.39 (7)	C8—C9—N2	131.4 (3)
O1—Pd1—O2	94.66 (7)	C4—C9—N2	106.9 (2)
O1^i^—Pd1—O2	85.34 (7)	N2—C11—C12	113.2 (2)
O3—Pd1—O2	91.39 (7)	N2—C11—H11A	108.9
O3^i^—Pd1—O2	88.61 (7)	C12—C11—H11A	108.9
O2^i^—Pd1—O2	180.00 (10)	N2—C11—H11B	108.9
O4—C1—O3	125.3 (2)	C12—C11—H11B	108.9
O4—C1—C2	120.2 (2)	H11A—C11—H11B	107.7
O3—C1—C2	114.5 (2)	O5—C12—O6	126.3 (3)
N1—C2—C1	112.7 (2)	O5—C12—C11	118.9 (2)
N1—C2—H2A	109.1	O6—C12—C11	114.8 (2)
C1—C2—H2A	109.1	C3—N1—C4	108.4 (2)
N1—C2—H2B	109.1	C3—N1—C2	126.0 (2)
C1—C2—H2B	109.1	C4—N1—C2	125.5 (2)
H2A—C2—H2B	107.8	C3—N2—C9	108.3 (2)
N2—C3—N1	110.3 (2)	C3—N2—C11	125.6 (2)
N2—C3—H3	124.8	C9—N2—C11	125.9 (2)
N1—C3—H3	124.8	Pd1—O1—H3W	115.2 (18)
C9—C4—C5	121.8 (2)	Pd1—O1—H4W	130.4 (18)
C9—C4—N1	106.0 (2)	H3W—O1—H4W	111 (2)
C5—C4—N1	132.3 (2)	Pd1—O2—H5W	118.6 (19)
C6—C5—C4	116.4 (3)	Pd1—O2—H6W	120.1 (19)
C6—C5—H5	121.8	H5W—O2—H6W	112 (2)
C4—C5—H5	121.8	C1—O3—Pd1	139.53 (17)
C5—C6—C7	121.5 (3)	H1W—O7—H2W	114 (2)

Symmetry codes: (i) −*x*+1, −*y*+2, −*z*.

### Hydrogen-bond geometry (Å, °)

**Table:**

*D*—H···*A*	*D*—H	H···*A*	*D*···*A*	*D*—H···*A*
O7—H1W···O4^ii^	0.822 (10)	1.985 (14)	2.756 (3)	156 (3)
O7—H2W···O5^ii^	0.821 (10)	1.937 (10)	2.747 (3)	169 (3)

Symmetry codes: (ii) −*x*+3/2, *y*−1/2, −*z*+1/2.
